# Supplementary figures and images for: Association between periodontitis and COVID-19 infection: a two-sample Mendelian randomization study
Source: PeerJ. 2023 Jan 25;11:e14595. doi: 10.7717/peerj.14595 (PMC9884046; doi:10.7717/peerj.14595)

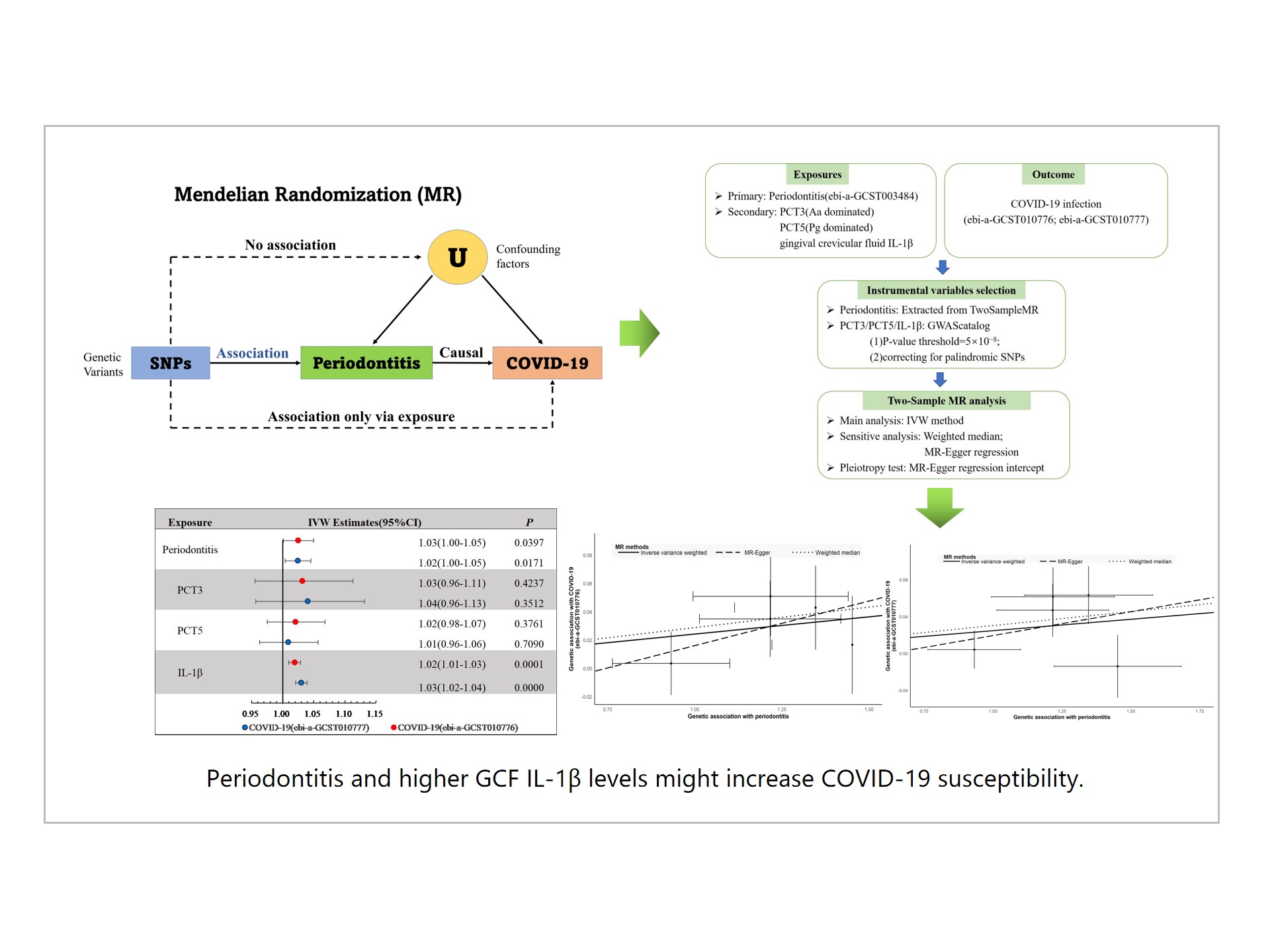

Supplement: Supplemental Information 1 [file peerj-11-14595-s001.jpg]
