# Supplementary material for: Association between periodontitis and COVID-19 infection: a two-sample Mendelian randomization study
Source: PeerJ. 2023 Jan 25;11:e14595. doi: 10.7717/peerj.14595 (PMC9884046; doi:10.7717/peerj.14595)
Supplement: Data S1 [file peerj-11-14595-s002.docx]

1. COVID
2. ebi-a-GCST010776

link：<https://gwas.mrcieu.ac.uk/datasets/ebi-a-GCST010776/>

PMID：32404885

Author：COVID-19 Host Genetics Initiative

DOI: 10.1038/s41431-020-0636-6

1. ebi-a-GCST010777

link：<https://gwas.mrcieu.ac.uk/datasets/ebi-a-GCST010777/>

PMID：32404885

Author：COVID-19 Host Genetics Initiative

DOI: 10.1038/s41431-020-0636-6

1. Periodontitis
2. Periodontal disease-related phenotype

GWAScatalogID：GCST003484

<https://www.ebi.ac.uk/gwas/publications/26962152>

PMID：26962152

Author：Offenbacher S, Divaris K, Barros SP et.al

DOI: 10.1093/hmg/ddw069

1. PCT3

GWAScatalogID：GCST008440

<https://www.ebi.ac.uk/gwas/publications/26962152>

PMID：26962152

Author：Offenbacher S, Divaris K, Barros SP et.al

DOI: 10.1093/hmg/ddw069

1. PCT5

GWAScatalogID：GCST008442

<https://www.ebi.ac.uk/gwas/publications/26962152>

PMID：26962152

Author：Offenbacher S, Divaris K, Barros SP et.al

DOI: 10.1093/hmg/ddw069

1. gingival crevicular fluid interleukin-1β [GCF IL-1β]

GWAScatalogID：GCST007542

<https://www.ebi.ac.uk/gwas/studies/GCST007542>

PMID：30206230

Author：Offenbacher S, Jiao Y, Kim SJ, Marchesan J, et.al

DOI：10.1038/s41467-018-05940-9
